# Dupilumab in acquired perforating dermatosis: A potential new treatment

**DOI:** 10.1016/j.jdcr.2022.08.013

**Published:** 2022-08-11

**Authors:** Musaed M. Alsebayel, Tariq Alzaid, Saud A. Alobaida

**Affiliations:** a Department of Dermatology, King Faisal Specialist Hospital and Research Centre, Riyadh, Saudi Arabia; b Department of Dermatopathology, King Faisal Specialist Hospital and Research Centre, Riyadh, Saudi Arabia

**Keywords:** acquired perforating dermatosis, dermatology, dupilumab, pruritus, treatment, APD, acquired perforating dermatosis, IL, interleukin

## Introduction

Acquired perforating dermatosis (APD) is an umbrella term that encompasses several disease entities that are characterized by transepidermal elimination of dermal connective tissue through the epidermis. These may include reactive perforating collagenosis, elastosis perforans serpiginosa, and perforating folliculitis.^1^ Various nomenclature has been used historically, including Kyrle disease and acquired reactive perforating collagenosis.^2^ The etiopathogenesis of the disease is not completely understood; however, it is hypothesized that interplay between several factors such as chronic scratching, reduced blood supply, overexpression of transforming growth factor β-3, and elevated serum and tissue levels of fibronectin are the main culprits. Diabetes mellitus and chronic kidney disease are the main risk factors; however, they can also occur in the setting of liver disease, hypothyroidism, and HIV.^2^^,^^3^

Dupilumab is a fully human monoclonal antibody that targets the α-subunit of the interleukin 4 receptor (IL-4Rα). This leads to the blockade of the signaling of IL-4 and IL-13, which in turn leads to inhibition of the T helper 2 cell response.^4^ It has been US Food and Drug Administration-approved for the treatment of several T helper 2 inflammatory conditions such as moderate-to-severe atopic dermatitis, asthma, and chronic rhinosinusitis.

The treatment of APD has been historically challenging. The most commonly used medications are topical steroids, antihistamines, and keratolytics. Systemic steroids, intralesional steroids, retinoids, doxycycline, allopurinol, and narrow-band UV-B therapy have all been used with variable success.^1^ In a recent study, dupilumab was used to treat APD in 2 patients with atopic dermatitis.^5^ To our knowledge, dupilumab has never been used as a monotherapy for the treatment of APD outside the setting of atopic dermatitis. Here, we present a case of a patient with APD who was treated with dupilumab with an excellent response.

## Case presentation

**Fig 1:**
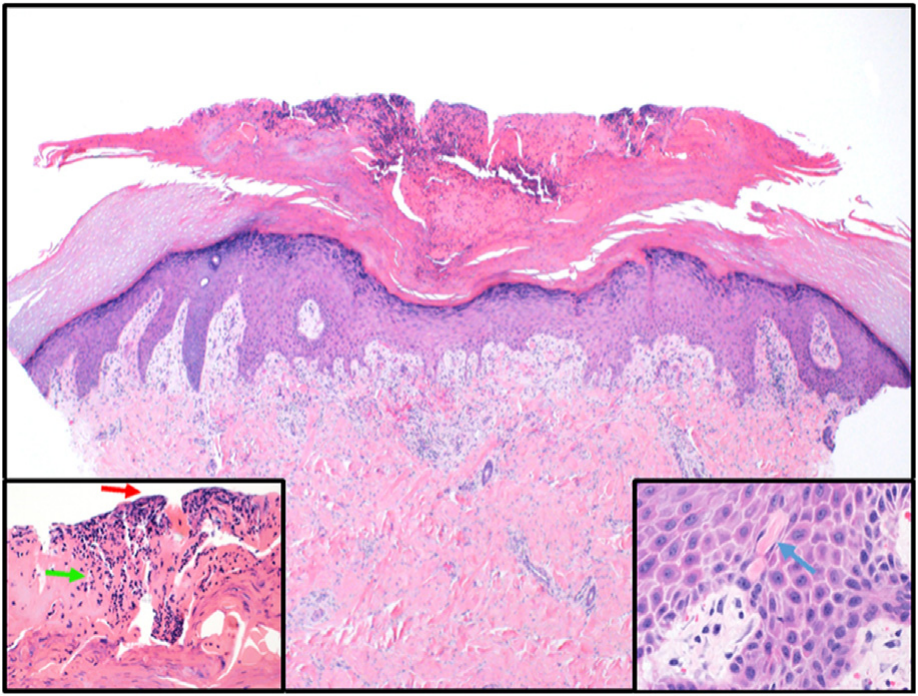
Hematoxylin-eosin–stained section showing skin with inflammatory crust (*green arrow*) and cellular debris (*red arrow*), underlined by depression in the epidermis with acanthosis. There are a few collagen bundles within the epidermis (*blue arrow*).

**Fig 2:**
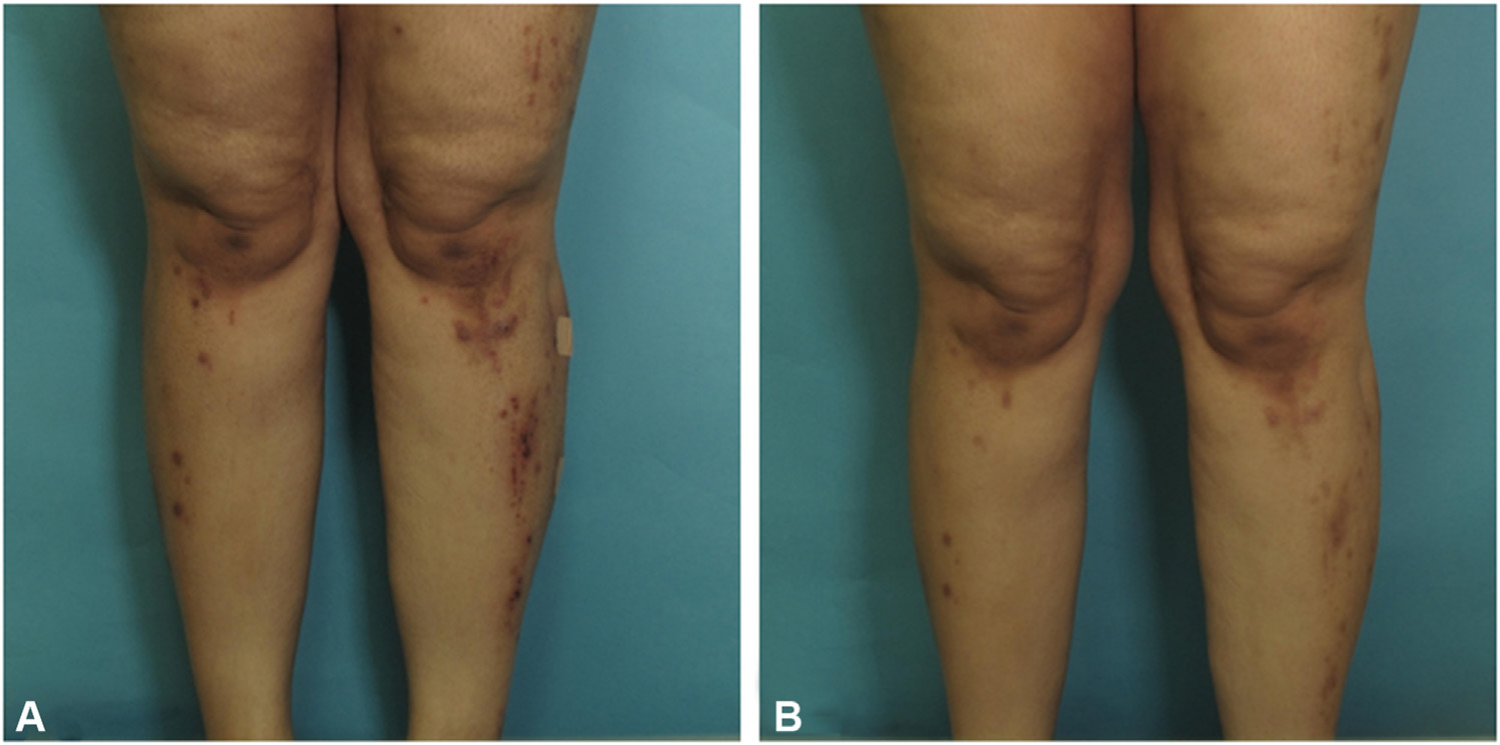
Multiple excoriated papules and nodules with central crust on the legs (**A**). Postinflammatory hyperpigmentation of previous lesions at 10-week follow-up (**B**).

The patient was a 20-year-old woman known to have Wilson disease status post liver transplantation 5 years prior. She presented to the dermatology clinic with a history of severe pruritus over the upper and lower portion of both the limbs. Her symptoms were interfering with her daily activities and making her unable to sleep at night. At the time of presentation, she was on oral tacrolimus 2.5 mg every 12 hours, lamivudine 100 mg once daily, and prednisone 20 mg once daily. Her symptoms started 2 months earlier and improved only slightly after starting prednisone 30 mg with tapering, which was prescribed by hepatology for high liver enzymes. On physical examination, she was found to have multiple hyperpigmented excoriated and umbilicated papules and nodules with central keratotic plugs on the upper and lower portion of both the limbs, sparing the face and trunk. Her laboratory reports were unremarkable except for a slightly elevated liver function test. A provisional diagnosis of APD was made, and the differential diagnosis included prurigo nodularis, arthropod bites, and hypertrophic lichen planus. A 4-mm skin punch biopsy was taken, which showed a neutrophilic crust, associated with cellular debris and underlined by acanthosis and depression in the epidermis. A few collagen bundles were noted within the epidermis, and these findings were consistent with a diagnosis of reactive perforating collagenosis (Fig 1). Given her complex medical history, traditional immunosuppressive therapy was not advised by her primary physician. She was started on dupilumab with a standard dose of 600 mg at day 0, then 300 mg every 2 weeks. The patient reported complete clearance of her pruritus within 2 weeks, and subsequently, her skin lesions started to subside. At the 10-week follow-up, the patient was off prednisone and postinflammatory hyperpigmentation was noted with no active lesions (Fig 2). She reported no medication side effects.

## Discussion

Perforating dermatoses are a group of disorders with transepidermal elimination of dermal connective tissue, that may be inherited or acquired. APD is a rare form of transepidermal elimination of collagen fibers that develop later in life. It has been suggested that APD is a reactive response to chronic rubbing and repetitive trauma to the skin.^6^ This is supported by the fact that when pruritus is controlled, lesions of APD resolve within a few months.^7^ In the case presented, the patient’s pruritus was presumably a result of her underlying liver disease. In a retrospective study, it was shown that liver disease is the third most associated disorder with APD, following diabetes mellitus and chronic kidney disease.^3^

Recent studies have shown that IL-4 and IL-13 act directly on the itch-sensory neurons, which leads to the promotion of chronic pruritus. In addition, IL-4 promotes neural hypersensitivity to several other factors such as histamine, chloroquine, IL-31, and cytokine-thymic stromal lymphopoietin, which are known to be strong pruritogens.^8^ Dupilumab is a fully human monoclonal antibody that directly binds to IL-4Rα of both IL-4 and IL-13. It has been shown to produce unprecedented efficacy in eliminating pruritus in patients with atopic dermatitis in multiple clinical trials.^9^ The rationale for the use of dupilumab, in this case, was for its antipruritic properties that in turn, would minimize skin trauma and the development of cutaneous manifestations. Its use as an antipruritic is successful in other chronic itch conditions such as prurigo nodularis, lichen planus, malignancy-associated pruritus, uremic pruritus, and pruritus of unknown origin.^7^ Ying et al^5^ showed in a recent study that dupilumab is an effective treatment for APD in 2 elderly patients in the context of senile atopic dermatitis. The use of dupilumab as a monotherapy for the treatment of APD has not been documented in the literature. The patient reported complete resolution of itch within 2 weeks of treatment, with subsequent resolution of her skin lesions. This successful experience may provide an additional line of treatment for severe APD.

## Conclusion

APD occurs in the setting of chronic itching. The treatment approach should aim to minimize pruritus and break the itch cycle. Dupilumab appears to be an effective treatment and should be considered for treatment-resistant APD.

## Conflicts of interest

None disclosed.
